# A Multidisciplinary Approach to Coronavirus Disease (COVID-19)

**DOI:** 10.3390/molecules26123526

**Published:** 2021-06-09

**Authors:** Aliye Gediz Erturk, Arzu Sahin, Ebru Bati Ay, Emel Pelit, Emine Bagdatli, Irem Kulu, Melek Gul, Seda Mesci, Serpil Eryilmaz, Sirin Oba Ilter, Tuba Yildirim

**Affiliations:** 1 Department of Chemistry, Faculty of Arts and Sciences, Ordu University, Altınordu, Ordu 52200, Turkey; aliyerturk@odu.edu.tr; 2 Department of Basic Medical Sciences—Physiology, Faculty of Medicine, Uşak University, 1-EylulUşak 64000, Turkey; arzu.sahin@usak.edu.tr; 3 Department of Plant and Animal Production, Suluova Vocational School, Amasya University, Suluova, Amasya 05100, Turkey; ebru.bati@amasya.edu.tr; 4 Department of Chemistry, Faculty of Arts and Sciences, Kırklareli University, Kırklareli 39000, Turkey; epelit@klu.edu.tr; 5 Department of Chemistry, Faculty of Basic Sciences, Gebze Technical University, Kocaeli 41400, Turkey; iremkulu@gtu.edu.tr; 6 Department of Chemistry, Faculty of Arts and Sciences, Amasya University, Ipekkoy, Amasya 05100, Turkey; 7 Scientific Technical Application and Research Center, Hitit University, Çorum 19030, Turkey; sedamesci@hitit.edu.tr; 8 Department of Physics, Faculty of Arts and Sciences, Amasya University, Ipekkoy, Amasya 05100, Turkey; serpil.eryilmaz@amasya.edu.tr; 9 Food Processing Department, Suluova Vocational School, Amasya University, Suluova, Amasya 05100, Turkey; sirin.oba@amasya.edu.tr; 10 Department of Biology, Faculty of Arts and Sciences, Amasya University, Ipekkoy, Amasya 05100, Turkey; tuba.yildirim@amasya.edu.tr

**Keywords:** SARS-CoV-2, COVID-19, cytokine storm, immunotherapy, vaccine development, in-silico research, small drugs, repurposing drugs, dietary supplements, natural products

## Abstract

Since December 2019, humanity has faced an important global threat. Many studies have been published on the origin, structure, and mechanism of action of the SARS-CoV-2 virus and the treatment of its disease. The priority of scientists all over the world has been to direct their time to research this subject. In this review, we highlight chemical studies and therapeutic approaches to overcome COVID-19 with seven different sections. These sections are the structure and mechanism of action of SARS-CoV-2, immunotherapy and vaccine, computer-aided drug design, repurposing therapeutics for COVID-19, synthesis of new molecular structures against COVID-19, food safety/security and functional food components, and potential natural products against COVID-19. In this work, we aimed to screen all the newly synthesized compounds, repurposing chemicals covering antiviral, anti-inflammatory, antibacterial, antiparasitic, anticancer, antipsychotic, and antihistamine compounds against COVID-19. We also highlight computer-aided approaches to develop an anti-COVID-19 molecule. We explain that some phytochemicals and dietary supplements have been identified as antiviral bioproducts, which have almost been successfully tested against COVID-19. In addition, we present immunotherapy types, targets, immunotherapy and inflammation/mutations of the virus, immune response, and vaccine issues.

## 1. Introduction

**Figure 1:**
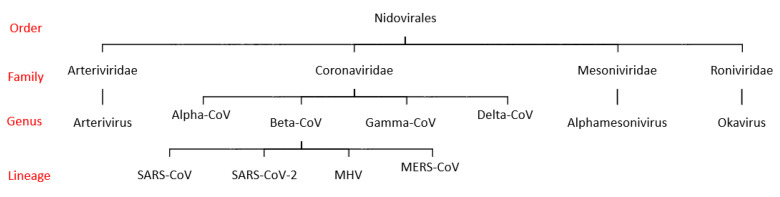
The current taxonomy of the order Nidovirales.

Over the past two decades, coronaviruses (CoVs) have been associated with significant disease outbreaks in East Asia and the Middle East. The severe acute respiratory syndrome (SARS) and the Middle East respiratory syndrome (MERS) began to emerge in 2003 and 2012, respectively. Previously, they were known to be important agents of respiratory and enteric infections of domestic and companion animals and to cause approximately 15% of all cases of the common cold. These viruses, a genus in the Coronaviridae family (order Nidovirales) (Figure 1), are pleomorphic and enveloped.

A new coronavirus that causes the coronavirus disease (COVID-19) recently emerged in the world in late 2019, known as severe acute respiratory syndrome coronavirus 2 (SARS-CoV-2), posing a global health threat [1]. The virus was first detected on 12 December 2019 in Wuhan City, Hubei Province, China. The World Health Organization (WHO) announced on 11 February 2020 that the current CoV-associated disease had been officially named COVID-19 [2].

CoVs belong to the Coronaviridae (subfamily Coronavirinae) family, whose members infect a wide variety of hosts, producing a variety of symptoms and diseases such as SARS, MERS, and currently COVID, which are all much more severe than the common cold and can be ultimately fatal. SARS-CoV-2 is considered one of the seven members of the CoV family that infects humans [3] and belongs to the same CoV lineage that causes SARS, but this new virus is genetically different [4,5]. Fan et al. predicted potential SARS or MERS-like CoV outbreaks in China following pathogen transmission from bats [6]. The emergence of new CoVs may have been made possible by the retention of more than one CoV in their natural hosts, which could support the possibility of genetic recombination [7]. The high genetic diversity and the ability to infect more than one host species are a result of high-frequency mutations in CoVs caused by the instability of RNA-dependent RNA polymerases (RdRp) together with higher rates of homologous RNA recombination [2,8]. Identifying the origin of SARS-CoV-2 and the evolution of the pathogen will make important contributions to disease surveillance [9], the development of targeted new drugs, and the prevention of other outbreaks [10].

From a considerable number (186) of pre-clinical developments worldwide, at least 87 in human clinical trials and 17 in emergency use have announced COVID-19 preventing vaccine candidates [11,12,13]. The messenger RNA vaccine, inactivated virus vaccine, DNA plasmid vaccine methodologies, and others were the start of COVID-19 prevention [14,15,16]. Other important treatment methods called immunotherapy can create an immune response against coronavirus.

Eighteen years have passed since the SARS outbreak in 2003 and, unfortunately, we still do not have a drug whose therapeutic efficacy is approved. Researchers and pharmaceutical establishments worldwide are currently working to develop an effective therapeutic to defeat this pathogen. Efforts on repurposing drugs continue as a more urgent solution to treat SARS-CoV infections. The drug repurposing approach is a work-saving, low-cost, safe, and effective treatment strategy for the pandemic period.

Many promising new compounds against COVID-19 have been synthesized, and computer-based methods have been used mainly to evaluate bioactivity. Fusion, which is the fusion of two substances so that there is no gap between them, is very important for the virus to enter the cell. Therefore, synthesis of new small-molecule fusion inhibitors is also an important research direction due to their shorter half-life and better bio-distribution than peptides. Although drug development studies have continued for years, research is promising on the design and development of new anti-COVID-19 molecules, based on existing information about the structure of the virus and its mechanisms of infection, replication, and mutation.

Other important and underlying topics of COVID-19 are (i) importance of the security of the food supply chain, (ii) food safety within the COVID-19 crisis during the lockdown period, (iii) awareness of food manufacturing/agricultural workers, and (iv) use of bioactive functional food.

In many cultures, natural products and traditional medicinal products are potential sources for the discovery of complementary new medicinal alternatives that prevent disease and are useful for their curative activities. The immunomodulatory and antiviral properties of functional food products as being low toxic, cheap, easily accessible, prophylactic, and supportive reagents have been demonstrated. In addition, the mechanisms by which these products interact with the virus in the host and their viral life cycle have been described. To reduce the risk of disease by strengthening the immune system, and accelerating the healing process of infected patients, we should include medicinal plants in our life, which we can easily access even if we are restricted to home [17].

## 2. Understanding the Mechanism and Structure of SARS-CoV-2

The Coronaviruses are the virus family with the greatest positive-polarity RNA genome. Having this genome causes less dependence on the host cell during replication of the virus. The replication occurs in the cytoplasm of the epithelial cells of the respiratory system and the gastrointestinal system. The term “corona” means crown in Latin. The virus takes its name from the crown-like structures in its frame [18,19]. 

Since the viral genome has positive polarity, the genome is used directly as a version, and various structural and nonstructural proteins are encoded. Firstly, genomic RNA is used as the version, and synthesis of the polyprotein 1a/1ab occurs, from which nonstructural proteins (nsp) are encoded to form a replication–transcription complex (RTK). After that, a range of interwoven subgenomic RNAs (sgRNA) are synthesized in discrete transcription style by RTK. The end of the transcription and accomplishment of the RNA in which the proteins will be encoded are provided from the transcription-regulator sequences located between the open-reading frames (ORF) [19,20,21]. The genome and the subgenome of a typical coronavirus have at least six ORFs. The first ORFs (ORF 1a/b) are two-thirds of the whole length of the genome and encode 16 nonstructural proteins (nsp 1–6). A frame slide of one-nucleotide between ORF1a and ORF1b ends with the production of two polypeptides: pp1a and pp1ab. These polypeptides are processed with 3CL^pro^ where the virus is encoded, or M^pro^ and one or two proteases and form 16 nsp [21,22,23].

At least four structural proteins are encoded from the other ORF regions, which are the remaining one-third of the genome: Spike (S), membrane (M), envelope (E), and nucleocapsid (N) proteins. In addition to these main structural proteins, the structural and accessory proteins specific to the virus such as the protein HE, the protein 3a/b, and the protein 4a/b are encoded in different coronaviruses [22,23,24]. These fractions suggest that nonstructural proteins are more conserved, and that structural proteins are more varied when adapting to new hosts. The mutation ratios in the replication of RNA viruses are much higher than those of DNA viruses, and the genome size of the RNA viruses is from 2 kb to 10 kb in general. The coronaviruses have the largest known RNA viral genomes with a length of about 30 kb. The persistence of the massive size of the genome is related to the characteristics of the RTK [24,25,26].

**Figure 2:**
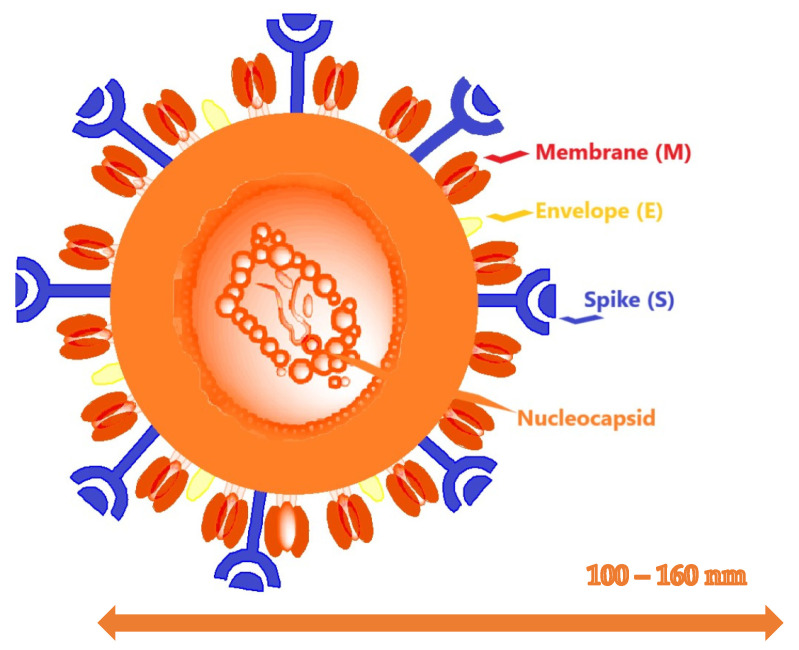
The SARS-CoV-2 virus structure.

The functions of the existing proteins are explained based upon the proteins of previously known coronaviruses. The functions of most of the nonstructural proteins in viral replication have been defined, but the functions of the remaining ones have not yet been defined. Four structural proteins have importance in the gathering of virions, the pathogenesis of the coronavirus infection, and in being the goal for developing new medicine (Figure 2, The schematic view of the coronavirus) [26,27,28].

**Figure 3:**
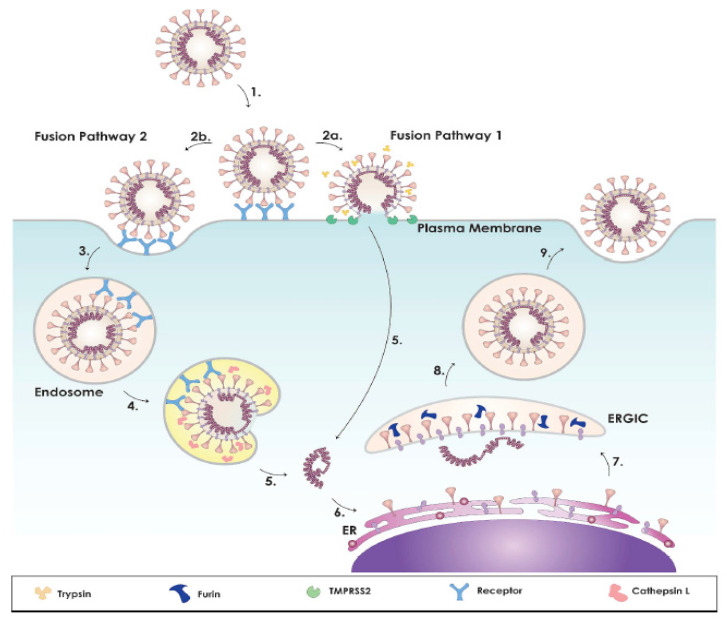
Model of coronavirus dual entry pathway. This model depicts the two methods of viral entry: Early pathway and late pathway. As the virus binds to its receptor (1), it can achieve entry via two routes: Plasma membrane or endosome. For SARS-CoV: The presence of exogeneous and membrane bound proteases, such as trypsin and TMPRSS2, triggers the early fusion pathway (2a). Otherwise, it will be endocytosed (2b, 3). For MERS-CoV: If furin cleaved the S protein at S1/S2 during biosynthesis, exogeneous and membrane-bound proteases, such as trypsin and TMPRSS2, will trigger early entry (2a). Otherwise, it will be cleaved at the S1/S2 site (2b) causing the virus to be endocytosed (3). For both: Within the endosome, the low pH activates cathepsin L (4), cleaving S2′ site, triggering the fusion pathway, and releasing the CoV genome. Upon viral entry, copies of the genome are made in the cytoplasm (5), where components of the S protein are synthesized in the rough endoplasmic reticulum (ER) (6). The structural proteins are assembled in the ER-Golgi intermediate compartment (ERGIC), where the S protein can be precleaved by furin, depending on cell type (7), followed by release of the virus from the cell (8, 9). For SARS-CoV-2: Studies currently show that SARS-CoV-2 can utilize membrane bound TMPRSS2 or endosomal cathepsin L for entry and that the S protein is processed during biosynthesis [29].

The S protein, which is in the shape of spikes on the viral envelope. The spike protein is responsible for receptor-dependent viral binding to the host cell and membrane fusion of the virus. The S protein is an important protein specifying the host cell tropism. The S1 loop of the S protein is responsible for binding to the host cell receptor and the S2 loop is responsible for membrane fusion. The S2 protein of 2019-nCoV has 93% similarity to bat-SL-CoVZC45 and bat-SLCoVZXC21. This similarity is about 68% for S1 protein. Both N and C terminal regions of the S1 loop can connect to the host cell receptor [27,28,29,30]. Although 2019-nCoV and SARS-CoV are in different bands, both viruses have 50 conserved proteins in their S1 proteins. The new coronavirus binds the angiotensin-converting enzyme 2 (ACE2) as a receptor, via S protein [31]. ACE2 is a receptor on the cell membrane surface in many human cells. ACE2 controls tension, inflammation, and wound healing, and plays an important role in biochemical pathways. ACE2 enters a cell by binding to the receptor binding region (RBD). SARS-CoV-2 makes contact after entering the cell and so damages the biological mechanisms controlled by the angiotensin biochemical pathway (Figure 3, [29]). Preventing SARS-CoV-2 from binding to ACE2 and entering the cell by ACE2 inhibitors is considered an important therapeutic tool. [32,33,34]. However, some current studies present conflicting reports about the entering mechanism of SARS-CoV-2 into the cell. Because of that, applying complementary treatment in addition to ACE2 inhibitors is important [35,36,37].

Transmembrane serine protease 2 (TMPRSS2) is a serine protease enzyme on cell membranes of the epithelial cells of the respiratory and gastrointestinal systems. The primary role of TMPRSS2 in SARS-CoV-2 biology is to prime the virus for membrane fusion via proteolytic cleavage. Its job is to cut the proteins in the region of serine amino acid, but its role in cells is not definitively known. However, it is known that it plays a role in some diseases such as prostate cancer and in the entering mechanism of SARS-CoV-2 into the cell. Heurich et al., (2014), showed that TMPRSS2 provides ACE2 activation of the coronavirus in entering the cell by cutting arginine and lysine amino acids from position 697 to 716 in ACE2 protein. Because of that, it is asserted that TMPRSS2 inhibitors can be used as prophylactic measures against SARS-CoV-2 infections [38]. Use of TMPRSS2 inhibitors with ACE2 inhibitors can decrease the entry of viruses to the cell.

Inhibitors of virus polyproteins: When the viruses enter the cell, they use the gene expression system of the host cell and produce their own proteins. RNA viruses primarily produce a large protein called polyprotein that contains all of the viral DNA, and then this protein is cut and transformed to virus proteins by viral or cellular proteases [39]. 3CL^pro^ is one of the enzymes, like PL^pro^, responsible for the processing of viral proproteins. For this reason, it is one of the key enzymes of viral replication. PL^pro^ is one of the enzymes providing coronavirus polyproteins to be processed in the host cell. Therefore, it is one of the main factors enabling the virus to spread in a patient’s cells. PL^pro^ inhibition is one of the main factors preventing the virus from spreading [40,41]. RNA-dependent RNA polymerases (RdRp) are responsible for the replication of viruses in the cell. The inhibition of this enzyme means the prevention of production of the nucleic acid of the virus. RdRp inhibitors have already been commonly used in the treatment of HIV, Zika virus, and Ebola infections. Since SARS-CoV-2 is also an RNA virus, RdRp could be effective against SARS-CoV-2 [42,43]. 

Coronaviruses encode four major structural proteins, namely, S, M, E, and N. Coronavirus S protein is a large, multifunctional class I viral transmembrane protein. S protein is on the virus surface on the viral envelope in the form of protrusions, binding to the receptor, and the virus holds onto the host cell by membrane fusion. The host cell is an important viral protein that determines its tropism. The S protein has S1 and S2 loops. Basically, the S1 protein enters the host cell receptor and prevents the S2 protein from binding to the membrane responsible for its fusion [27,28,44]. Another structural protein, the M protein, together with the N protein, are E proteins that play a very important role in virus formation and release. They have three transmembrane compartments and their virions (virion = complete virus particle) increase the membrane loop, bind to the nucleocapsite, and provide stabilization of the nucleocapsid protein. The E protein puts together the viral parts and has an assembly role in virus release and pathogenesis play. Its role in pathogenesis is not fully known, although E protein oligomerization causes ion channel formation. The viral genome contains two fields through different mechanisms that can connect. Replication of viral RNA plays a role in the regulation of its transcription [43,44,45,46]. 

After SARS-CoV-2 enters respiratory epithelial cells, it causes an immune response with inflammatory cytokine production accompanied by weak interferon (IFN) response. By activating the nuclear factor kappa B (NF-kB) pathway through one of the virus genetic material toll-like receptors (TLR), TLR3, TLR7/8, and TLR9, pro-inflammatory cytokines such as interleukin (IL)-6 and tumor necrosis factor α (TNFa) provide synthesis. The natural immune system is first degree in the immune response developed against viruses.

The cells involved in the innate immune response (macrophages, monocytes, dendritic cells, neutrophils) that recognize infectious agents on its surface and cytoplasm are binding receptors. Called a “pattern recognition receptor (PTR)”, these receptors are also expressed in cells that are the target of SARS-CoV-2. Major PTRs include toll-like receptors (TLR), NOD-like receptors (NLR), and RIG-I-like receptors (RLR). Binding of these molecules to their receptors triggers the signal transduction mechanism within the cell and the synthesis of inflammatory or anti-inflammatory cytokines.

In addition, when the infectious agent is a single-stranded RNA virus, the genetic material binds to TLRs (TLR7/8, TLR-9) found in endosomes, stimulating Type-I interferon synthesis (IFNa, IFNb), which plays an important role in defense against viruses [47]. After these cytokines are secreted from the cell, they bind to their own receptors (IFNAR-I, IFNAR-II) and activate the pathway that enables the synthesis of antiviral proteins. The role of antiviral proteins is to create new virions inside the cell to prevent an antiviral state. Therefore, in order to limit COVID-19, it is necessary to produce sufficient amounts of Type-I IFN in the early period. Otherwise, viruses multiply and spread to all tissues. 

So that antiviral response is delayed, virus replication is increased and virus-related. It has been shown that the cytopathic effect spreads gradually in the tissue [48,49,50]. Early infection, delay, or no occurrence of Type-I IFN response during the period of innate immunity causes its components to come into play. Thus, to control the infection, neutrophils, monocytes, macrophages, lymphocytes, and NK cells begin to accumulate and an exaggerated immune response occurs. Hyperinflammation, a condition that is triggered by infection in the body and has severe inflammatory responses in the body, is a process where viral proteins are found in target cells (epithelial cells, endothelial cells, macrophages), NOD (nucleotide-binding oligomerization domain), LRR (leucine-rich repeat), and pyrin domain-containing protein 3 (NLRP3). The inflammasome complex provides IL-1b and IL-18 synthesis by stimulating it [51]. Increased cytokines (cytokine storm) in the early and advanced stages of infection cause local and systemic inflammation. While they contributed to a host’s ability to tolerate and survive, they also stimulate adaptive immunity [48,49,50]. 

In a recently published study, CC chemokine ligand (CCL) 2, CXC chemokine ligand (CXCL) 2, CCL8, CXCL1, CCL3L1, and IL-33 in bronchoalveolar lavage samples of patients with COVID-19, and IP-10 in peripheral blood, tumor necrosis factor superfamily-10 (TNFSF10), tissue inhibitor of metalloproteinases-1 (TIMP1), complement (C) 5, IL-18, amphiregulin, neuregulin1, and IL-10 were detected. It has been suggested that IL-10 is an indicator of hyperinflammation and cytokine storm [52,53]. Although many cytokines and chemokines are secreted in these cases, the high level of IL-6 in plasma has been associated with a poor prognosis and risk of death [54]. 

Hyperinflammation develops during severe COVID-19 infection and initiates the prothrombotic process by causing cell activation and dysfunction. In this process, platelets, coagulation factors, and innate immune cells play a role in clot formation because they are in constant interaction with each other. This is called immunothrombosis (thromboinflammation). Although this is beneficial in preventing the spread of the pathogen and providing structural support to the endothelium, it contributes to the development of acute respiratory distress syndrome (ARDS) by causing uncontrolled and widespread immunothrombosis and widespread microangiopathy [55].

## 3. Immunotherapy and Vaccine

**Figure 4:**
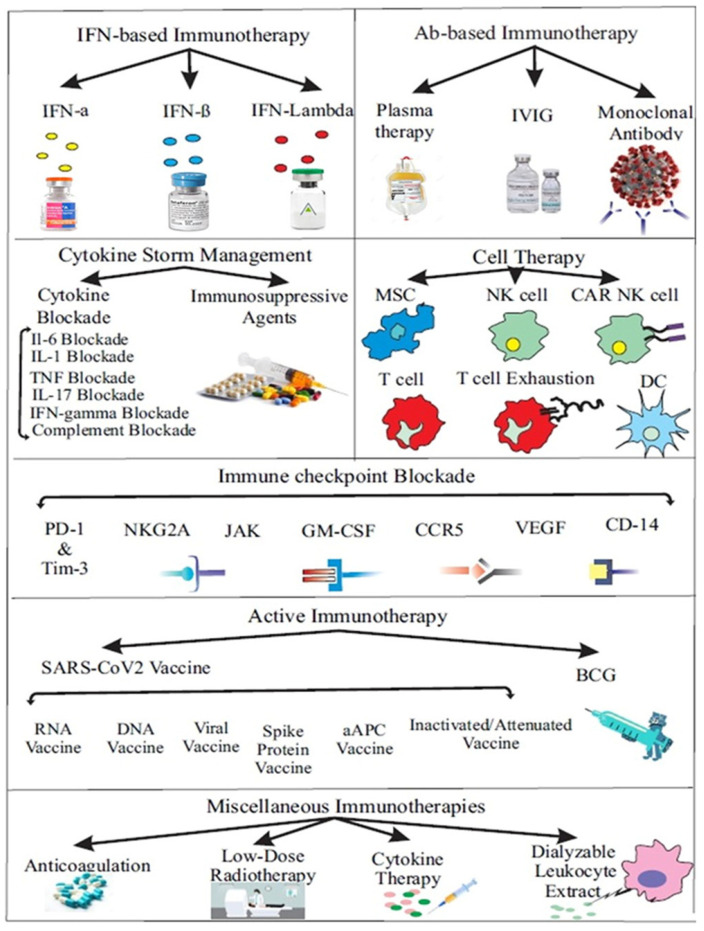
Development of therapeutics targeting immune responses for COVID-19 through interferon- and antibody-based therapies, cytokine storm management, anti-inflammatory radiotherapy, and cell therapy [61].

Immunotherapy treatment induces an immune response to the disease or increases the immune system’s resistance to diseases such as HIV and cancer [56,57,58]. Severe inflammation caused by an inadequate and dysfunctional antiviral immune response is one of the challenges in COVID-19. The development of therapeutics that target immune responses via active immunotherapy, interferon-based immunotherapy, antibody-based therapies, cytokine storm management, anti-inflammatory radiotherapy, and cell therapy could improve the clinical outcomes of COVID-19 patients [59,60] (Figure 4, [61]). Widely used anticoagulation, checkpoint inhibitors, vaccines, cytokines, interleukin, interferon, CAR-T cell therapy, monoclonal antibodies, and colony-stimulating factors can play an important role in immunotherapy [62,63]. Immunotherapy initiatives for 2019-nCoV contain the polyclonal antibody for plasma therapy, the polypeptide hormone for immunoglobulins, T cell maturation, Angiotensin-converting enzyme 2 (ACE2) immunoadhesin, and monoclonal antibody against interleukin-6. Applications used for SARS-CoV include viral vectors, nanoparticles, inactivated viruses, and DNA and monoclonal antibodies, which are also promising for the treatment of 2019-nCoV [64]. Although coronaviruses have an exonuclease gene product that provides higher accuracy during genome replication, antibody escape mutations remain a concern. Mutations that affect antibody neutralization could occur and become fixed as the virus circulates during the pandemic. A cocktail of monoclonal antibodies, rather than a single agent, may decrease the likelihood of neutralization escape [65,66].

It is now well known that pulmonary pneumocytes are the most extensive lung cells infected with SARS-CoV-2. The cytotoxic effects of the virus in pneumocytes lead to stimulation of the inflammation-mediated release that triggers immunity. Proinflammatory cytokines such as TNF, IFN, IP-10, monocyte chemotactic protein-1 (MCP-1), and chemokines are produced in alveolar macrophages to create an inflammatory immune response against the virus. These pro-inflammatory cytokines and chemokines are released into the blood and blood monocytes and T lymphocytes create an inflammatory response in the lung. Usually, immunity can eradicate the virus; however, if insufficient immune response or respiratory tract hyperinflammation occurs, severe respiratory failure can be seen in cases of COVID-19. Severe pulmonary inflammation also increases capillary leakage that can cause [67,68]. Therefore, the immune response to SARS-CoV-2 and the severity of inflammation are two major factors in Covid-19 patients [69]. Immunotherapy has shown significant results in the treatment of many diseases such as cancer and viral infections. Plasmacytoid dendritic cells (pDCs) increase antiviral immunity between adaptive and innate immune reactions [70]. Antigen-specific interactions distinguish between DCs (Dendritic cells) and T cells that induce an adaptive cellular immune response. Activated CD8+ T cells stimulate the aggregation of DCs (XCR1 + DCs) expressing the XCR1 chemokine receptor located in lymph nodes. Hence, better co-functioning between pDCs and XCR1 + DCs increases the maturation of XCR1 + DCs and antigen cross-presentation. Antigen activated CD8+ T cells are modulated by collecting more DC in the antigen recognition domain [71]. C-type lectin, which is called dendritic cell-specific intercellular adhesion molecule-grabbing nonintegrin (DC-SIGN), found in peripheral mucosa and expressed on the DC surface, has been found to play an important role in the binding of many viruses to host cells. In order to assess trans infection of targeted T cells, C-SIGN is important, despite not being a receptor in SARS-CoV infection. Antibodies raised against DC-SIGN can inhibit DC infections and become targets for designing new therapies [72].

To control SARS-CoV-2 infection, suppression of Type I interferon-producing pathways can be prevented, viral infection can be targeted, and the inflammatory response can be controlled with immunomodulatory approaches. If active immunity is induced by viral vaccines, it may inhibit the spread of the virus into populations, especially in patients with severe comorbidities. According to the World Health Organization, there are 186 (+2) worldwide pre-clinical developments, of which 87 (+4) are in human clinical trials and 17 are in the WHO EUL/PQ (Emergency Use Listing/Prequalification) evaluation process [73,74,75]. Many studies are conducted by studying different vaccine mechanisms using live attenuated vaccines, inactivated virus vaccines, subunit, viral vector, DNA, and mRNA-based vaccine technologies [76,77]. Inactivated virus vaccines containing killed virions have been tested, and their efficacy and safety have been demonstrated in all-phase studies against SARS-CoV coronaviruses [77,78]. Viral particles of inactivated virus vaccines have lost their pathogenicity and do not show a risk of disease in immunocompromised individuals [77,79]. However, inactivated vaccines may produce lower amounts of antigen-specific antibodies and therefore require booster vaccines for the efficacy and protection of the vaccines. Unlike inactivated and live attenuated vaccines containing complete pathogens, subunit vaccines may contain antigenic fragments and adjuvants to increase immunogenicity. Nevertheless, subunit vaccines provide short-term immunity by inducing only immune memory immunity and generally produce less potent CD8+ responses. 

Viral vector-based vaccines use vaccine vectors to express antigens and transfer them to host cells. The vaccine vector neutralizes the antigen before it delivers it, which can reduce the effectiveness of the vaccine. Before the vaccine vector delivers the antigen, it can neutralize the vaccine’s effectiveness, and carcinogenic effects may occur due to possible integration of the viral genome into the host genome. DNA vaccines provide transcription of an antigen and adjuvant that can mimic infection, such as live viruses. The advantage of DNA vaccines is that they have temperature stability with the induction of humoral and cell-mediated immune responses [77,78]. However, DNA vaccines may not be effective by inducing weak cytotoxic and humoral immunity. They can also activate oncogenes and increase cancer risk by involving the integration of viral DNA into the host genome. Although similar technologies are used in mRNA vaccines with DNA vaccines, there is no risk of integration into the host genome [77,79]. In addition, they can cause expression of antigens that can model infection with a live virus. They may have a relatively unstable structure compared to DNA viruses and elicit reactogenicity or an inflammatory response in vaccination [77,78]. 

The mRNA vaccines Pfizer/BioNTech (Germany) reported 95% efficacy (94% over 65 years) and Moderna (USA) reported 95% [77]. Of the viral vector vaccines, the World Health Organization reported Oxford Uni/AstraZeneca (UK) 90–62% efficacy, and Sputnik V (Russia) 92% efficacy [80]. It has been reported hat the inactivated virus vaccine Sinovac (China) has 83.5% efficiency in Turkey, 65% in Indonesia, and 50% in Brazil [81]. More research is needed to determine the efficacy of available immunotherapeutic treatments for host viral interaction and SARS-CoV-2 [61,80,82,83]. Various side effects may occur as a result of the use of immunotherapy, depending on the type of treatment. Side effects such as flu-like symptoms, loss of appetite, diarrhea, fever, weakness, nausea, muscle aches, and vomiting can be seen. Side effects such as redness, bruising, or bleeding are usually of short duration, but patients may require hospitalization if they develop serious problems [84,85].

## 4. Computer Aided Drug Design

Computational and simulation approaches, based on classical physics and quantum mechanical principles, offer theoretical approaches to structure-activity from small chemical systems to macro-scale biological molecules through algorithms. Drug design development and understanding the molecular basis of SARS-CoV-2 can be modulated by computational methods. Researchers have benefited from various computational methods, especially virtual screening (molecular docking/scoring, quantitative structure activity relationship (QSAR), etc.) as well as ligand-based design (classical and De-Novo design) in understanding the molecular basis of SARS-CoV-2, which has spurred rapid drug design. 

A considerable part of SARS-CoV-2-focused modelling studies includes DFT-based analyses, which offer approaches related to the concept of electron density. Determination of molecular geometries, with the most stable minimum energy of ligands having the potential to bind to the active protein sites of targets, enables calculations to give more successful results in the molecular docking process. DFT-based optimized geometry allows for the study of frontier molecular orbitals that play an important role in understanding the interaction mechanisms between drugs and their receptors in determining the hit ligands against SARS-CoV-2 [86,87,88,89,90]. The difference between HOMO (highest-energy molecular orbital occupied by electrons) and LUMO (lowest-energy molecular orbital not occupied by electrons) energy values plays a critical role in determining the chemical reactivity, charge transfer capabilities, and bioactivity potential of molecular systems [91,92,93,94,95]. The ligand with a narrower HOMO-LUMO energy gap will also have a higher potential to inhibit the active site of the protein [96,97]. In a study conducted with a DFT-based approach, the calculated energy gap (ΔE) for United States Food and Drug Administration (FDA)-approved Favipiravir was 2.14 eV, and 5.54 eV for another antiviral agent, Ribavirin [98]. In this work, the authors interpreted the wider bandgap calculated for Ribavirin as being due its molecular structure having several hydrophilic interactions that can facilitate binding with receptor. They stated that these types of hydrophilic interactions may affect the binding affinity of small-scale drugs to receptors. 

Researchers are interested in some reactivity descriptors such as ionization potential, electron affinity, global chemical softness and hardness, electronegativity, chemical potential, and electrophilicity index in focused studies against SARS-CoV-2. Known clinical trials drugs—Baloxavir, Chloroquine (CQ), Avigan (Favipiravir), Plaquenil, Oseltamivir, Remdesivir, Arbidol, and Sofosbuvir—have been studied based on various reactivity parameters. The higher antiviral activity potential of Aviga (Favipiravir) than other drugs has been associated with its lower chemical potential value, which is explained by both inter-and intramolecular hydrogen bond capacity of Favipiravir compounds [99]. In a study on hypertensive drugs targeting the coupling of ACE2 in the host and S protein in SARS-CoV-2, the antiviral activity of Ramipril, with higher chemical softness and electrophilicity index values, was determined to be more promising than other drugs [100]. Molecular electrostatic potential (MEP) maps are one of the tools used in the docking of ligands with SARS-CoV-2 proteins depending on their charge density to predict the regions that will provide the most appropriate geometry and score value [100,101,102]. MEP maps created with computational methods estimate sites that are especially sensitive to electrophilic and nucleophilic interactions of molecular structures before molecular docking [103,104,105]. Software programs that researchers frequently use include AMBER, AVOGADRO, DMOL3, GAUSSIAN, HYPERCHEM, JAGUAR, and MOE [106,107,108,109,110,111,112,113,114].

The drug design process has useful multi-step tools to avoid time-consuming and expensive investigation [113]. This process has two different approaches: Structure-based or ligand-based computer-aided drug design [114,115,116,117,118,119]. Both methods are suitable for SARS-CoV-2 and lots of results have been obtained for use in clinical treatment. The virtual screening method in drug design and development against SARS-CoV-2 is the physical high-throughput screening (HTS) to find the lead compound. The preparation of the target protein and also receptor–ligand complex structures must be detailed for HTS of drug design. So far, too few three-dimensional structures have been well-defined and determined [87,120,121,122].

Until now, it has been evident that multiple-calculation strategies are necessary to find an effective and magic drug for COVID-19, such as molecular dynamics, De-Novo design, docking, homology modelling, and ADMET (absorption, distribution, metabolism, excretion, and toxicity). The most commonly employed drug targets are the antiviral drugs, secondary metabolites such as flavonoids, alkaloids, etc., anticancer drugs, and different enzyme inhibitor drugs [123,124,125,126,127,128,129].

The molecular dynamics approach, based on classical Newtonian mechanical equations, is used to observe two important steps in the computational analysis of SARS-CoV-2: (i) Defining virus protein conformation of the ligand-binding domain (the molecular structures of proteins are elucidated with X-ray diffraction, electron microscopy, and neutron diffraction before dynamic properties are identified) and (ii) calculating the stability and flexibility of protein and ligand and the process of entry and exit of ligands to protein binding sites [130,131]. Molecular dynamics (MD) simulation is obtained in the most widely used software, such as AMBER, GROMACS, GROMOS, CHARMM, CHARMm, LAMMPS, DL-POLY, NAMD, and DESMOND [132,133]. 

Many options have been successfully explored for the target structures including SARS-CoV-2 M^pro^ in complex with N3 inhibitor (6LU7), non-structural proteins such as papain-like protease/deubiquitinase inhibitors (3E9S), chimeric receptor-binding domains complexed with ACE2 (6VW1), and E protein pentameric ion channels (5 × 29) [134].

Yang and colleagues were the first to use in-silico methods upon the SARS-CoV-2 M^pro^ N3 complex structure not only for identification of the target, but also to determine the substrate-binding pocket with Glide software. Using HTS approaches to improve the repurposing of approved drugs and natural products, Yang and co-workers screened 10,000 members of the library using fluorescence resonance energy, in which seven candidates, Ebselen, Carmofur, Disulfiram, Shikonin, Tideglusib, PX12, and TDZD8, have been found to be greatly effective in M^pro^ inhibition activity. To test the applicability of the computer-aided drug design, when an in vitro antiviral activity test was realized, Disulfiram > Tideglusib showed the strongest antiviral effect so that molecular docking results are paralleled, and a binding energy of −46.16 and −61.79 kcal/mol with iFit-dock and Glide (v8.2) software, respectively, was obtained [87].

In the 6LU7-targeted SAR and MD study, Gromiha and colleagues discovered the synergistic effect of Lopinavir, Oseltamivir, and Ritonavir. Using the Autodock program, the binding energy was calculated as −4.1 kcal/mol for lopinavir, −4.65 kcal/mol for Oseltamivir, and −5.11 kcal/mol for Ritonavir, and MD simulation (in 100 ns) of the root-mean-square deviation (RMSD) was stable around 2 Å, 1 Å, and 3 Å, respectively, via Amber software [135].

Tiwari offered a different perspective by De-Novo design on 11 antiviral drugs using retrosynthetic analysis approaches that target the interaction of the S glycoprotein of SARS-CoV-2 with ACE2. A docking study was performed targeting RBD-ACE2 (6VW1) with antiviral molecules using Glide’s Extra Precision. As a result, the Gibbs free energy binding was −40.8 kcal/mol (Lopinavir) and −38.7 (Ribavirin) kcal/mol. Similarly, MD analysis and calculated RMSD results showed that ascorbate and Ribavirin have the best interaction with 6VW1. Ligand-based drug design via De-Nova approaches was generated as ascorbate, Ribavirin, Lopinavir, and Hydroxychloroquine (HCQ) to obtain VTAR-01. These compounds were re-designed to have better ADMET properties than 11 antiviral drugs selected as drug candidates [136].

**Figure 5:**
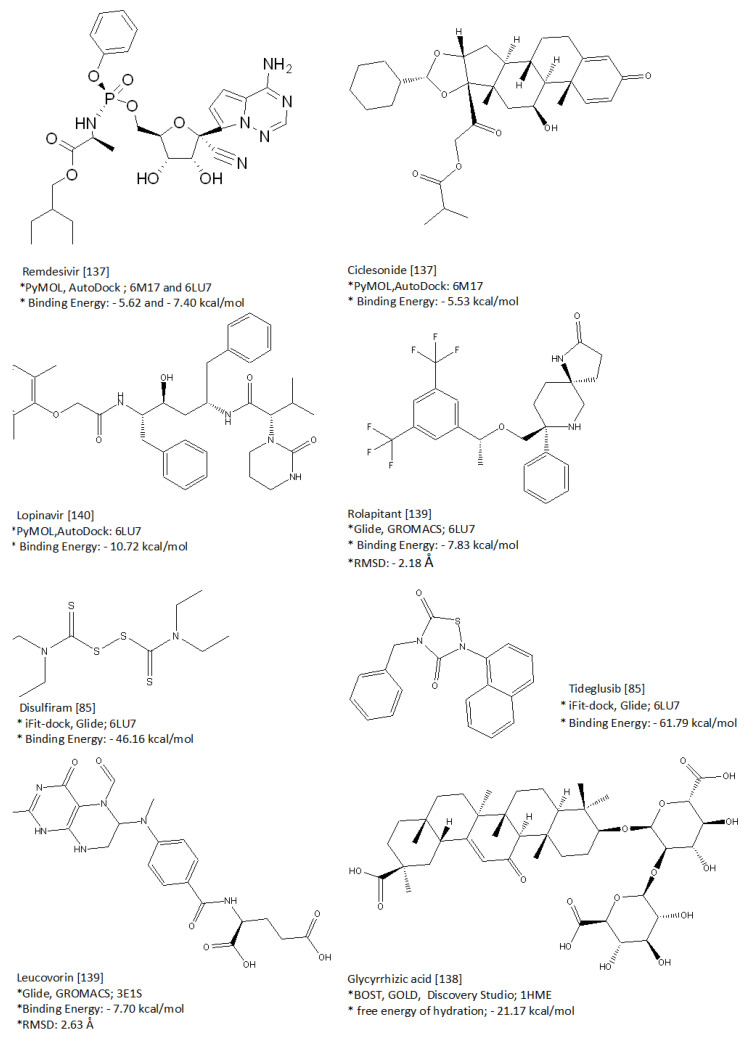
Calculation results of the highlighted potential drugs of SARS-CoV-2.

The computer-aided methods provide beneficial results that can expand and guide not only the discovery of new drugs, but also repurposing drugs. The focus of this part of the review is to discuss recent advances in computer-aided drug design against COVID-19 using methods such as QSAR, MD, and ADMET. Some of the highly specific and sensitive pharmacophore models, which were determined by these methods, are summarized in Figure 5 [137,138,139,140]. Targeted NSP proteins including M^pro^, RdRp, and PL^pro^ were analyzed regarding their docking with FDA-approved 1615 ligands in the ZINC15 database with AutoDock Vina, Glide, and rDock software [lit3]. An antiemetic drug named rolapitant was suggested in this work, depending on the results of the RMSD and binding energy values. To examine the flavonoid compounds that may inhibit 6LU7 M^pro^ in the complex with the inhibitor N3, computational analysis was generated, and kaempferol and quercetin were found to have high binding performance [140]. 

## 5. Therapeutics for Covid-19 Treatment

While several different types of vaccines have currently been approved worldwide, there is still no specific effective treatment or prevention available against COVID-19. Together with the injustices in vaccine supply and availability, the importance of finding an influential therapeutic treatment increases. It is worth mentioning that discovering new sovereign molecules for COVID-19 is a long, costly, and complex process. Due to the prolonged pandemic process and the lack of a suitable treatment as of yet, the drug repurposing approach comes to the fore (Table 1). The fact that the side effects of the drugs used in this approach are known and clinical studies and regulation studies have been conducted provide rapid results [141,142,143,144]. Each of the drugs used in the treatment are effective at different stages of the virus’s life cycle. It is necessary to understand the mechanism of action of therapeutics due to levels of illness in order to optimize treatment for people with COVID-19 [145]. However, in some cases, the opposite results can occur. For instance, in the first wave of COVID-19, chloroquine (CQ) and hydroxychloroquine (HCQ), which are well-known antimalarial drugs, were recommended as a primary treatment option for COVID-19 [146,147,148]. However, later in the pandemic, well-designed randomized controlled trials confirmed that the CQ/HCQ regimen does not provide any clinical benefit for COVID-19 patients [149].

### 5.1. Antivirals

Molnupiravir: Molnupiravir (EIDD-2801/MK-4482) is a prodrug of the ribonucleoside analog β-D-N4-hydroxycytidine (EIDD-1931 [NHC]), which is phosphorylated intracellularly to the active 5′-triphosphate [150]. Molnupiravir has been demonstrated to have good tolerability and pharmacokinetics in clinic studies for COVID-19 and is currently under investigation in Phase II and III clinical trials after Merck licensed the compound from Ridgeback Biotherapeutics (USA) (NCT04405570, NCT04405739, NCT04575597, and NCT04575584) [150,151]. Based on the latest findings on clinical development of molnupiravir from Merck and Ridgeback Biotherapeutics, phase 3 trials will only proceed for non-hospitalized patients. For hospitalized patients, Molnupiravir has not demonstrated any benefit [152]. 

Remdesivir: Remdesivir (RDV, GS-5734, brand name Veklury, Gilead Science, USA) is a cyano-substituted adenosine analog, a prodrug form of the monophosphate adenosine analog GS-441524. It is a RdRp blocker that acts by inhibiting the viral replication of nucleic acid via bond formation with the active site of RdRp [153,154,155]. Remdesevir was first identified to treat Ebola virus and then discontinued due to being less effective than other therapies [156,157]. Remdesevir has also been used as an antiviral drug to treat other known RNA viruses, like MERS-CoV and SARS-CoV [158,159]. Remdesivir was the first United States Food and Drug Administration (FDA)-approved drug for the treatment of COVID-19 patients on 22 October 2020. Before FDA approvement, on July 2020 remdesivir received a conditional approval in Europe [142,160,161]

Favipiravir: Favipiravir (6-fluoro-3-hydroxy-2-pyrazinecarboxamide) (FPV, brand name Avigan, Toyama chemical, Japan)) is an oral pyrazinecarboxamide derivative and guanine analogue that directly halts the transcription by inhibiting the RdRp of RNA viruses [159,162,163,164]. The antiviral drug Favipiravir was approved for all subtypes of influenza in many countries such as Japan, China, and Russia. Favipiravir was also accepted for the treatment of Ebola virus infection [165,166,167].

In studies conducted for SARS-CoV-2, FPV has been shown to effectively inhibit the virus in Vero E6 cells [159,168]. After phase-3 clinical trials, favipiravir is suggested as a potential candidate drug in mild to moderate to COVID-19 [169].

Lopinavir/Ritonavir: The Lopinavir–ritonavir combination (known as Kaletra^®^, Abbott, USA) is a protease inhibitor. This combination has been used in the treatment of various viruses such as HIV and also used against COVID-19 in clinical trials as an emergency treatment in some countries [143,170,171,172,173,174]. In the early stage of COVID-19, this combination could reduce the viral load and improve the disease symptoms [175]. Currently, many clinical trials are proceeding in many countries for this combination drug [143,149,176].

### 5.2. Anti-Inflammatory Compounds

Colchicine (Takeda Pharmaceuticals, USA) is an anti-inflammatory compound used to treat gout and Behçet disease [177,178,179,180]. Additionally, it has antiviral properties against dengue and Zika viruses [181]. Moreover, colchicine may affect HIV viral load [182]. There are some clinical trials on the effectiveness of colchicine against COVID-19. The initial results showed that colchicine alone or in combination with other drugs (Lopinavir/Ritonavir, Dexamethasone, or Hydroxychloroquine) had a significant mortality benefit (84% vs. 64% survival) and less need for supplemental oxygen [183,184,185].

Naproxen (Perrigo Company, USA) has both anti-inflammatory and antiviral properties [186]. It is used to treat rheumatoid arthritis, psoriatic arthritis, osteoarthritis, and gout [187]. One of the clinical trials revealed that the larithromycin–naproxen–oseltamivir combination reduced the influenza virus. Naproxen is also in clinical trials for the treatment of SARS-CoV-2 virus. A recently reported clinical trial treating COVID-19 patients with a combination of Azithromycin (250 mg/daily), Prednisolone (25 mg/daily), Naproxen (250 mg twice a day), and Lopinavir/Ritonavir (200/50 mg g tablets, two times/12 h) showed effective results [188,189].

### 5.3. Antibacterial Compounds

Azithromycin (Pfizer, USA) is a macrolide-type antibiotic that is used to treat many bacterial infections. It is widely used in chronic lung diseases, infections of the sinuses, ears, throat, and skin [190]. 

Azithromycin disrupts bacterial growth by interfering with their protein synthesis. It has anti-inflammatory and antiviral effects (Zika, Ebola, rhinovirus, influenza viruses) [191,192]. Alone and in combination with other medications, it is currently under clinical trials for the treatment of COVID-19. Azithromycin alone did not show antiviral activity [193,194,195]. However, the combination of Hydroxychloroquine at 5 µM with Azithromycin at 5 µM and 10 µM significantly inhibited viral replication. There are several effective trials of Azithromycin used in combination with other drugs such as Nitazoxanide, Ivermectin, and Beta-lactams [196,197,198].

Teicoplanin (Sanofi-Aventis, France) is a semi-synthetic glycopeptide antibiotic. It is usually used in the prevention and treatment of serious infections caused by Gram-positive bacteria [199]. It has shown efficacy against various viruses, such as influenza virus, Ebola virus, hepatitis C virus, and human immunodeficiency virus (HIV), as well as the coronaviruses such as SARS-CoV and MERS-CoV [200,201]. Mechanistic studies that were performed by Zhang et al. showed that Teicoplanin blocked virus entry, in particular by inhibiting the activity of cathepsin L. Furthermore, they showed that Teicoplanin also inhibits the entry of SARS-CoV-2 [202]. However, more clinical trials should be done.

### 5.4. Corticosteroid Compounds

Dexamethasone (Pfizer, USA) is an anti-inflammatory synthetic adrenal corticosteroid and is used to treat rheumatic diseases, skin diseases, allergies, asthma, and lung diseases. In cancer patients undergoing chemotherapy, Dexamethasone is usually given against some of the side effects of antitumor treatments [203,204]. Dexamethasone is usually given to hospitalized COVID-19 patients requiring oxygen therapy. In a recent report, it was shown that in patients hospitalized with COVID-19, the use of Dexamethasone resulted in lower 28-day mortality among those who were receiving either invasive mechanical ventilation or oxygen alone at random, but not among those receiving no respiratory support [205,206,207].

Currently, inhaled corticosteroid (ICS) therapy trials are also being investigated in COVID-19 treatment. This therapy in chronic obstructive pulmonary diseases reduces expression of the SARS-CoV-2 entry receptor ACE2. This effect may therefore contribute to altered susceptibility to COVID-19 in patients with chronic obstructive pulmonary disease [208]. 

Methylprednisolone (Pfizer, USA) is a synthetic corticosteroid. It has anti-inflammatory and immunomodulating properties. It is used to treat lupus, arthritis, asthma, allergic reactions, skin, kidney, lung diseases, and immune system disorders [209,210]. Methylprednisolone is currently under clinical trial for the treatment of COVID-19 patients. It was successful in treating COVID-19-associated pneumonia in one of the trials [211,212]. A recent study showed that in hospitalized patients suffering from COVID-19 pneumonia, the administration of 2 mg/kg per day of intravenous methylprednisolone compared to treatment with 6 mg/day of dexamethasone led to a reduction in the hospital length of stay and need for mechanical ventilation [213].

### 5.5. Antiparasitic Compounds

Nitazoxanide (Romark, USA) is a nitrothiazole benzamide compound. It is active against various parasites, Gram-positive and Gram-negative bacteria, and viruses [214]. It is used in the treatment of influenza and other respiratory viruses, Hepatitis B, Hepatitis C, HIV, and MERS-CoV [215,216]. Currently, many clinical trials are being examined for using Nitazoxanide alone or in combination with Azithromycin, Ivermectin, or Hydroxychloroquine to manage patients with COVID-19. The ability of protecting the lungs and preventing associated multi-organ damage makes nitazoxanide a promising candidate for reuse in COVID-19 [196,217].

Ivermectin (Merck Sharp & Dohme Corp., USA) is a semi-synthetic anthelmintic agent. It is used to treat various types of parasitic infections in veterinary and human medicine [218]. Recent investigations have shown that Ivermectin has antiviral activity against some viruses such as West Nile, Zika, Influenza A, and HIV-1 [219]. Antiviral activity of ivermectin alone or in combination with other drugs towards COVID-19 is under research in many trials [220,221]. Based on a recent report, multidrug therapy with Ivermectin, Azithromycin, Montelukast, and Acetylsalicylic acid (TNR4) improved recovery and prevented risk of hospitalization and death among ambulatory COVID-19 cases [222].

### 5.6. Anticancer Compounds

Camostat mesylate (Towa Pharmaceutical, Japan) is an inhibitor of the enzyme TMPRSS2. Therefore, it is a potential antiviral drug against COVID-19. It is used to treat some forms of cancer, chronic pancreatitis, and postoperative reflux esophagitis [223,224]. Clinical trials are currently ongoing, and the recent reports reveal that Camostat mesylate is effective against COVID-19 [225]. Hoffmann et al. showed that the virus can use TMPRSS2-related proteases for S protein activation and that these enzymes were also blocked by Camostat mesylate. Furthermore, they showed that the Camostat mesylate metabolite GBPA exhibits reduced ability to block enzymatic activity of purified, recombinant TMPRSS2 and was rapidly produced under cell culture conditions [226].

Gemcitabine (Eli Lilly and Company, Indianapolis, IN, USA) is a chemotherapy medication used to treat several types of cancer. It is classified as an antimetabolite [227]. It has been notified that the combination of Gemcitabine with Decitabine reduced HIV infectivity [228]. After that, another study showed that the DNA synthesis inhibitor Gemcitabine has antiviral effects against MERS-CoV and SARS-CoV [229]. In addition, a new study has shown that the combination of Gemcitabine and Oxysophoridine is effective against COVID-19 [230]. 

Imatinib (Novartis, USA) is a tyrosine kinase inhibitor with antineoplastic activity. It is used to treat a number of types of cancer [231]. There are various studies on whether this drug will be effective against COVID-19. A recent study showed that Imatinib is able to inhibit this virus. Another in-silico study showed that the use of Imatinib in combination with Losartan may be effective in patients infected with SARS-CoV-2 [232,233].

Tamoxifen (Sandoz, Australia) is an antineoplastic nonsteroidal selective estrogen receptor modulator (SERM) of the triphenylethylene group. It is extensively used to treat and prevent breast cancer [234]. A number of studies have shown that it potentially has antifungal, antimicrobial, antiparasitic, and antiviral activities. It was reported that Tamoxifen is active against human immunodeficiency virus (HIV), Hepatitis C virus (HCV), and Ebola virus (EBOV) [235]. Tamoxifen may be used as immunotherapy against COVID-19 due to its capability to modulate NK cells activity and reduce viral replication [236]. However, it is stated in the literature that tamoxifen may increase the risk of thrombosis [237].

### 5.7. Antipsychotic and Antihistamine Compounds

Chlorpromazine (Sanofi, UK), Fluphenazine (Pai Pharmaceutical, USA) and Promethazine (Sandoz, Australia) are phenothiazine derivative compounds. They are used to treat behavioral disorders and they have antipsychotic, anxiolytic, antiemetic, antiviral, and immunomodulatory effects, together with the inhibition of clathrin-mediated endocytosis [238]. They have shown antiviral activity against MERS-CoV and SARS-CoV viruses. Some previous studies showed that they reduce viral replication of MERS-CoV and SARS-CoV possibly through the inhibition of clathrin-mediated endocytosis [239,240]. The clinical trials of phenothiazine derivatives are ongoing.

Fluvoxamine (Solvay Pharmaceuticals, Belgium) is an antidepressant, and it is a selective serotonin reuptake inhibitor (SSRI) [241]. A recent double-blind, randomized, preliminary study of adult outpatients with symptomatic COVID-19 showed that patients treated with Fluvoxamine, compared to those treated with placebo, had a lower likelihood of clinical deterioration over 15 days [242]. Another study showed that with the use of Fluvoxamine for early treatment of COVID-19, the incidence of hospitalization was 0% with Fluvoxamine and 12.5% with observation alone. At 14 days, 0% of Fluvoxamine-treated people had persistent residual symptoms compared to 60% among people who opted for no therapy [243].

### 5.8. Antihypertensive Compounds

Losartan (Merck Sharp & Dohme Limited, UK) is a potassium salt of the aromatized, negatively charged tetrazole compound. It is used to treat high blood pressure [244]. It is an angiotensin II receptor type 1 (AT_1_) antagonist. Recent reports recommend that AT_1_R blockers such as losartan may work to alleviate the symptoms of COVID-19 [245,246,247]. 

### 5.9. Antidiabetic Compounds

Metmorfin (Merck Serono Limited, UK) is used to in the treatment of type 2 diabetes [248]. Bramente et al. point out that Metformin is associated with reduced mortality in women with obesity or type 2 diabetes who were admitted to hospital for COVID-19. They showed that metformin could be widely distributed for the prevention of COVID-19 mortality, since it is safe and inexpensive [249].

In another study, Wang et al. showed that metformin in primary care does not influence susceptibility to COVID-19, COVID-19 related mortality, or all-cause mortality. However, glycemic control should continue to be the best advice for patients with diabetes, especially if rates of COVID-19 rise [250].

### 5.10. Immunosuppressive Compounds

Sirolimus (Pfizer Europe MA EEIG, Belgium) is a macrolide compound and was originally developed as an antifungal agent. However, subsequent studies showed that sirolimus has remarkable antitumor and immunosuppressive activities. It is used to prevent organ transplant rejection and treat lung disease [251]. Research has continued on the effectiveness of sirolimus against COVID-19 [252]. Sirolimus’ function as an mTOR inhibitor could help to inhibit COVID-19 virus replication [253]. However, more trials need to be done.

Cyclosporin (Sandoz, Australia) is an immunosuppressive drug. It is used to treat inflammation in rheumatoid arthritis and to prevent the rejection of organ transplants [254]. Recent reports showed that cyclosporin inhibits SARS-CoV-2 virus replication, and it is in clinical trials, alone and in combination with other drugs, for the treatment of COVID-19 at present [255,256].

### 5.11. Immunomodulators

Anakinra (Sobi Inc., Stockholm, Sweden) is a drug that has been proven to be effective in rheumatoid arthritis and auto-inflammatory diseases. Anakinra, a recombinant receptor antagonist for IL-1, is one of the cytokine-blocking agents used for COVID-19 treatment. Some of the clinical trials have shown that Anakinra is effective in reducing clinical signs of hyperinflammation in critically ill COVID-19 patients [257,258]. 

Bamlanivimab (Eli Lilly and Company, USA) is a monoclonal antibody, which was developed for treatment of COVID-19 [259]. It can be used alone or with combination with another monoclonal antibody etesevimab against COVID-19. Based on the latest reports, treatment with combination of bamlanivimab and etesevimab provides significant reduction in COVID-19 viral load [260]. 

Baricitinib (Eli Lilly and Company, USA) is a drug for the treatment of rheumatoid arthritis. It acts as an inhibitor of janus kinase (JAK) and blocks the JAK1 and JAK2 [261]. Baricitinib can be used alone or in combination with antivirals in COVID-19 treatment as well. The highest reported efficacy of baricitinib was against COVID-19 pneumonia, mostly in patients receiving oxygen support without invasive mechanical ventilation [262,263].

Bevacizumab (Roche and Genentech, Switzerland-USA) is a monoclonal antibody used in the treatment of several types of cancer and an eye disease. It inhibits vascular endothelial growth factor A (VEGF-A), and it might be beneficial for treating COVID-19 patients [264]. Bevacizumab shows clinical efficacy by improving oxygenation and shortening oxygen-support duration in recent clinical trials [265].

Sarilumab (Sanofi and Regeneron, France-USA) is a monoclonal antibody medication against IL-6 (Interleukin 6). IL-6 is a cytokine that plays an important role in immune response. It is used in the treatment of rheumatoid arthritis [266]. When used in specific doses, sarilumab can be effective against COVID-19 [267,268]. However, more trials are needed.

Tocilizumab (Roche and Genentech, Switzerland-USA) is a monoclonal antibody used against IL-6 (Interleukin 6). It is used in the treatment of rheumatoid arthritis and systemic juvenile idiopathic arthritis [269]. Tocilizumab is also used in the treatment of COVID-19 [270]. There are still some trials ongoing. 

Lenzilumab (Humanigen Inc., Burlingame, CA, USA) is also a monoclonal antibody. It targets cytokine granulocyte macrophage colony-stimulating factor (GM-CSF), and it also has potential immunomodulating activity [271]. In COVID-19 treatment, a recent study showed that lenzilumab can improve survival without the need for mechanical ventilation and is more beneficial than steroids and/or remdesivir [272].

Casirivimab/Imdevimab is an experimental medicine developed by the American biotechnology company Regeneron Pharmaceuticals. It is an artificial “antibody cocktail” designed to produce resistance to the SARS-CoV-2 coronavirus responsible for the COVID-19 pandemic [273]. Clinical trials are still ongoing.

## 6. Synthesis of New Molecular Structures against COVID-19

An overall search of the therapeutic approaches to cure SARS-CoV-2 infection reveals that repurposing drugs is the remedies mostly used. However, when looking at overall mortality, length of hospital stays, and the start of ventilation time, these drugs appear to have little or no effect on hospitalized COVID-19 patients and they show undesired side effects [274]. Thus, there is an urgent need to develop new lead compounds as we know that new interventions would likely require years. 

Some promising research towards the synthesis of new molecular structures to overcome COVID-19 has been carried out. According to studies on synthesis since the beginning of the COVID-19 pandemic, researchers have mainly focused on two approaches to target the virus when designing the new molecules. The first is the inhibition of SARS-CoV-2 M^pro^, also called the 3CL^pro^ enzyme, which is important for viral replication, and the second is fusion inhibition via binding to the N-terminal RNA binding domain (NTD) of the N-protein of the virus. A survey of the literature reveals that it is possible to group the synthesized new molecular structures into four groups: (i) Aromatic/nonaromatic heterocyclic compounds bearing aliphatic/aromatic substituents, (ii) isoquinolines, (iii) lipopeptides, and (iv) peptidomimetic α-ketoamides. Peptidomimetic inhibitors are known for the treatment of several diseases such as cancer, autoimmune diseases, and diabetes, and they also have structural diversity and unique modes of action [275]. It is not surprising that heterocyclic compounds are one of these groups as they are present in many drugs. The research on newly synthesized structures with potent anti-COVID-19 activity reveals different approaches to measure the bioactivity against SARS-CoV-2. These approaches include structure–activity relationship (SAR) works, in vitro/in vivo assays and in-silico methods covering molecular docking studies, DFT calculations, QSAR studies, overlaying of X-ray structures of inhibitors onto the active site of SARS-CoV-2 M^pro^, ADME, and drug-likeness studies via pharmacokinetics/pharmacodynamics (PK/PD) properties.

**Figure 6:**
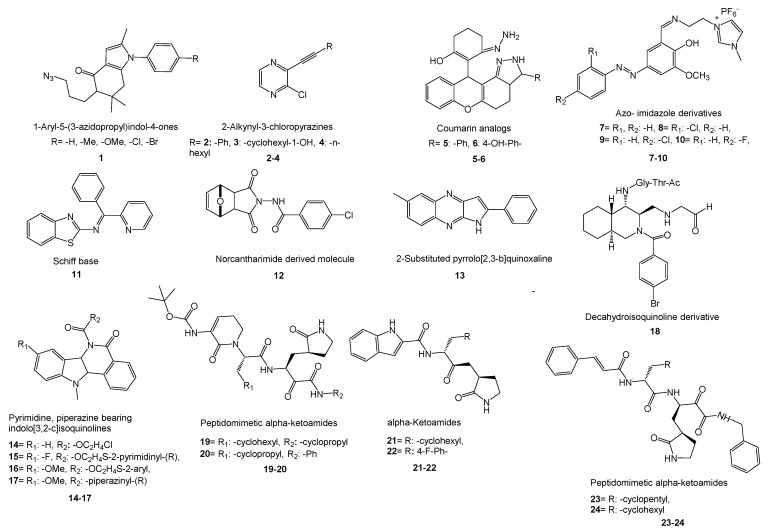
New molecules with anticipating anti-COVID-19 properties.

Because of the importance of this subject, we want to present recent literature on the synthesis of new molecules anticipating anti-COVID-19 properties and these works are summarized below (Figure 6).

(i) Domínguez-Villa and co-workers reported the synthesis of five new azidopropylindol-4-ones (**1**) as a potential inhibitor of SARS-CoV-2 3CL^pro^ [276]. Molecular docking studies, ADME/Tox profile, and drug-likeness works showed favorable properties of the compounds with low toxicity. Therefore, fine bioavailability levels were foreseen. Research on copper-catalyzed sonochemical synthesis of 2-alkynyl-3-chloropyrazines showed binding affinity of the compounds onto the NTD of N-protein of SARS-CoV-2 [277]. The researchers followed SAR, molecular docking, and ADME studies to show the compounds are prospective ligands for SARS-CoV-2. Three new compounds (**2**–**4**) were found as potential agents for further studies. In a promising work, novel coumarin analogs and some natural coumarin analogs were investigated to inhibit SARS-CoV-2 M^pro^ via molecular docking and PK studies of ADME and drug-likeness [278]. Among the synthetic coumarin analogs, two compounds (**5**,**6**) revealed good binding energy inhibition potential. A recent work on the inhibitory action of azo- imidazole derivatives against SARS-CoV-2 M^pro^ presented four new synthesized compounds (**7**–**10**) as promising agents by comparing the efficacy of the molecules with FDA-approved and some repurposed antiviral drugs using molecular docking and ADME research [279]. Ahmed et al. synthesized three new Schiff bases as potential SARS-CoV-2 3CL^pro^ inhibitors [280].

Molecular docking studies and DFT calculations with antibacterial results identified one of the Schiff bases (**11**) as the most potent agent. The results of research on the synthesis of a series of new norcantharimide-derived molecules revealed that these molecules (**12**) showed physicochemical properties that could be considered as orally active drug candidates, while docking studies indicated that they exhibited good theoretical affinity for M^pro^ [281]. Chemboli and co-workers reported research on the synthesis of 2-substituted pyrrolo[2,3-b]quinoxalines as potent cytokine storm attenuating agents in COVID-19 [282]. Most of the compounds showed reasonable and significant inhibition of TNF-α and acceptable toxicity in vitro as a result of the structure–activity study. It was found that compounds with free NH groups (**13**) are more effective than their *N*-sulphonyl analogs. Moreover, several compounds were found to be promising for their binding affinities via docking onto the NTD of N-protein of SARS-CoV-2.

(ii) Novel pyrimidine, piperazine-bearing indolo[3,2-c]isoquinolines were synthesized as potent COVID-19 M^pro^ inhibitors by Verma’s group [283]. Molecular docking studies exhibited good interactions of 4 compounds (**14**–**17**) with 6LZE (COVID-19) and 6XFN (SARS-CoV-2) at active sites. In another work on the synthesis of isoquinoline derivatives, molecular docking and in vitro studies revealed that the decahydroisoquinoline scaffold (**18**) is a good hydrophobic moiety to interact with S2 site of SARS 3CL^pro^ [284].
(iii) Research covering the synthesis of a series of lipopeptides demonstrated that these peptides are potent coronavirus fusion inhibitors [285]. Cytotoxicity studies, in vitro cell–cell fusion assays, and in vivo mouse infection studies demonstrated that one peptide drug is the most potent fusion inhibitor against SARS-CoV-2 and can be used in an inhalation formulation to treat patients.
(iv) Zhang and co-workers reported the synthesis of peptidomimetic α-ketoamides and PK properties of optimized SARS-CoV-2 M^pro^ inhibitors (**19**,**20**). They revealed a pronounced lung tropism showing that it is suitable for use by the inhalative route [286]. In another work, new α-ketoamides were synthesized, and two of them (**21**,**22**) showed satisfactory SARS-CoV-2 3CL^pro^ inhibitory activity [287]. The aldehyde groups were covalently linked to cysteine145 of 3CL^pro^ and showed in vivo PK properties. Additionally, in vivo toxicity studies with (**21**) in SD rats and hunting dogs revealed no significant toxicity in either group. In another study, designed peptidomimetic α-ketoamides were synthesized against M^pro^ of coronaviruses and 3CL^pro^ of enteroviruses [288]. The researchers found two near-equipotent inhibitors (**23**,**24**) by testing the compounds against recombinant proteases, in viral replicons and virus-infected cell cultures. They showed once again that structure-based approaches in the development of broad-spectrum antivirals are a powerful tool.

A valuable review on the subject showed the possible targets to beat the SARS-CoV-2 virus guiding and stimulating medicinal chemists to synthesize new anti-COVID-19 molecules [289]. These routes are: (i) The interaction of the S glycoprotein of SARS-CoV-2 with heparan sulfate proteoglycan (HSPG) on the host cell surface. This group may include converters of S1 and S2 subunits, hydrophobic small molecules, heparin/heparin sulfate (HS)-based oligosaccharides, or HS mimetics. Protease inhibitors especially can hinder conjunction with the ACE2 receptor. (ii) Binding of the S protein to the ACE2 receptor. This offers the possibility to use soluble ACE2 and S1 subunit-based peptides or peptidomimetics or anti-ACE2 antibodies. In particular, endocytosis or cathepsin L inhibitors can decrease the effectiveness of virus-cell conjunction. (iii) The proteolytic separation of S glycoprotein. This step can be blocked by inhibitors of serine or cysteine proteases. Similarly, inhibitors of proteases included in endocytosis (e.g., cathepsin L) will decrease viral infectivity. (iv) Various enzymes and non-enzymatic proteins take part in viral replication, such as E, M proteins, and RNA-dependent RNA polymerase (RdRp). It is a suitable stage for small molecule discovery. v) Lastly, the virus is set free from the host cell surface. This part can also be blocked by protease or heparinase inhibitors that contribute to the process. For instance, HS mimetics united with a mixture of protease inhibitors can prevent virus outflow.

## 7. COVID-19 Pandemic and Food: Safety and Functional Food Components

COVID-19, the most significant biological disaster the world has faced in the 21st century, has become more than a health issue and has threatened reliable food supply and the sustainability of the food supply chain more than ever [290,291]. In this brief summary, the scientific studies on food safety and supplements positively affecting the immune system during the pandemic are described. Thus, it is important for the supply chain not to be broken to sustain the quality of life during and after the pandemic. Regarding the safety of food, the supply of consistent and sufficient food and the economic accessibility of individuals to food has strategic importance [292,293]. Movement restrictions, imposed to prevent the spread of SARS-CoV-2 infection, have heavily affected food-related activities, including food production, processing, and distribution. The quick evolution of the pandemic and the arrival of the second wave caused distrust, inability to see into the future, and stocking of food in fear of scarcity, which caused the food stock in markets to temporarily run out [294]. A study conducted in the USA compared the grocery shopping behaviors of consumers before and after the pandemic. The results showed that the highest priority is market shopping, and that food is the top priority after drugs [295]. In a food-related statement published by the FDA in 2020, it was stated that there is no information showing that SARS-CoV-2 can be transmitted through food or food packaging [296]. Although coronavirus strains have been determined to be stable at low (<70 °C) and freezing temperatures, it has been reported that the contamination of viruses to food can be prevented by hygiene and food safety practices [297]. However, several different cases were identified where food, especially meat products, was found to be suitable as a vector. Viral infection was detected in a meat processing plant in Germany [298], seafood processing facilities in China [299], on the inner walls of packages, and on containers carrying frozen shrimp [300] and frozen chicken wings [301]. Therefore, consuming raw or undercooked products should be avoided. Raw meat, raw milk, or raw animal tissues and organs should not be consumed and contact with cooked or uncooked foods and thus cross contamination should be prevented [296]. Therefore, it is always important to ensure good hygiene practices such as frequently washing hands and surfaces while handling and preparing food, keeping raw meat separate from other foods, cooking food at the right temperature, and cooling it quickly [294,297,302].

Smart packaging technology where human contact is minimal [303], smart freezing and thawing technology [304], electronic nose [305], smart hyperspectral imaging system (HIS) technology [306], and applicable technologies such as artificial intelligence (AI) should be considered within these businesses.

It has been predicted that the integration of technology and innovation into food processes will contribute to the reduction of virus transport and in turn stop the rate of spread. Although technology is being integrated into systems, the therapeutic properties of foods that are included in individual consumer’s habits and diet lists are now being studied. Nutrition plays a very important role in promoting long-term health and curing chronic diseases. Proper nutrition is critical for an effective immune system and both malnutrition and over nutrition can negatively affect immune responses. Numerous news sites and reports on websites as well as social media platforms for the prevention of SARS-CoV-2 infection, nutrition, and strengthening the immune system convey the message that dietary supplements or certain foods can prevent the spread of the new coronavirus [307,308,309].

Many functional foods, phytochemicals, probiotics, and vitamins have been shown to have beneficial effects in strengthening the immune system [310]. Propolis has been determined to block PAK1 when using 1 mL drops per day for each 10 kg of body weight for individuals in the treatment of COVID-19 [311]. In the study where its effects on influenza infection treatment were examined, consumption of 10g/day dietary fiber was associated with low mortality rates caused by communicable respiratory diseases [312]. All tested polyphenols have an inhibiting effect on SARS-CoV protease with an IC50 ranging between 30.2 and 233.3 μM [311]. Quercetin regulates immunity when taken at 500 mg and 1000 mg a day. Curcumin has been reported to be able to directly inhibit the entry of SARS-CoV-2 into target cells in terms of direct antiviral activity [313]. Melatonin, which can be used as a potential adjuvant for COVID-19, can help boost the immune system, inhibit inflammation, and regulate oxidation stress [314]. Probiotic microorganisms can modulate the immunity system [315]. All immune cells have Vitamin D3 receptors. Vitamin D not only has antiviral effects, but also reduces inflammation that damages the lining of the lungs and decreases pro-inflammatory cytokine concentrations, as well as increasing anti-inflammatory cytokine concentrations [316]. Vitamin C is frequently used in the treatment of influenza and colds, and its use is recommended as 7.5 mL daily for children aged 1–2 years, 25 mg/5 mL for children aged 3–4 years, and 10 mL for children aged 5–6 years [317]. Vitamin A and vitamin C have synergistic immunological functions [318]. Furthermore, 13.3 mg of Zinc gluconate consumption has been associated with quickly diminishing symptoms of colds, fewer days of coughing, and less voice loss, headache, and nasal obstruction [319]. Many micronutrients such as zinc, selenium, copper, and iodine modulate the Dual oxidase (DUOX) system to increase its oxidative killing power against viruses. Many nutritional supplements such as vitamin C, glutathione, *N*-acetylcysteine (NAC), organic sulfur compounds, and medicinal herbs can reduce inflammatory responses caused by viruses due to their antioxidant properties. [320].

Researchers agree that social isolation, hygiene, and strengthening of the body’s immune system are the most effective methods to combat the COVID-19 epidemic.

## 8. Potential Natural Products against COVID-19

In this section, traditional natural products used throughout human history in the treatment of viral infections and to strengthen the human immune system, especially medicinal plants with proven supportive efficacy in the treatment of SARS and MERS, have been summarized as promising potential herbal resources for COVID-19. In a global sense, the potentials of natural products against this infection, which interrupts the daily life of all humanity, have been evaluated according to their effectiveness in inhibiting different main stages of the viral cycle of host-SARS-CoV-2 interaction. In Table 2, some natural products that stand out with their activities of inhibiting the major steps of SARS-CoV-2 in the host were evaluated with different methods and parameters.

In the research in this area, it has been observed that flavonoids, a class of natural polyphenolic compounds commonly found in plants, have a striking potential against the virus. It has been stated that this phytochemical, which has various pharmacological properties, especially antioxidant, antiviral, anti-inflammatory, and antineoplastic activities, exhibits inhibitory properties in almost every step in the viral cycle of SARS-CoV-2 [321]. Baicalin (EC_50_: 12.5 μg/mL), a bioactive glycosylated flavonoid found especially in *Citrus* species, while showing the most effective antiviral activity as an ACE2 inhibitor against the prototype SARS virus growing in the fetal Rhesus Kidney-4 (FRhK-4) cell line [322,323], an in silico study made for TMPRSS2 of SARS-CoV-2 has also shown that it acted as a natural inhibitor. Again, baicalin and baicalein (baicalin aglycon; selectivity index (SI): 19 µM and 118 µM) inhibited 3CL^pro^ with a potency close to CQ (EC_50_: 1.13 µM and SI: 88.61 µM) in the in vitro test and fluorescence resonance energy transfer protease test on Vero E6 cells contaminated with SARS-CoV-2 [324]. In a study in China where detailed analysis of all proteins encoded by SARS-CoV-2 genes was performed, it was suggested that hesperidin could prevent the virus from entering the cell because it overlaps significantly with the ACE2 interface [325]. It has been noted that caffeic acid, the strongest phenolic bioactive component in the structure of *Sambucus formosana* Nakai, with many biologic potentials including antiviral activity, blocks the adhesion of HCoV-NL63 to the cell surface regardless of the cell type used [323]. According to a study in androgen-sensitive human prostate (LNCaP) adenocarcinoma cells, kaempferol (flavonol) suppressed TMPRSS2 by 49.14% and 79.48% at 5 and 15 μM, respectively [326]. Results of various structure–activity studies showed Hirsutenone (diarylheptanoid) isolated from *Alnus japonica*, curcumin (polyphenol) from *Curcuma longa*, xanthoangelol E and xanthoangelol F (prenylated chalcones) isolated from *Angelica keiskei*, and two flavonoids psoralidin and isobavachalcone in ethanolic extract of *Cullen corylifolium* (L.) seed as candidates for SARS-CoV PL^pro^ [322,327]. Quercetin, another important flavonoid for SARS-CoV-2, is both a potent ACE2 inhibitor [324] and a potent 3CL^pro^ inhibitor (ΔG: −6.6 kcal/mol), while at the same time having low cytotoxicity (EC_50_ = 83.4 μM; CC_50_ = 3.32 μM) [323]. Nevertheless, the concentration required for inhibition could not be reached with oral administration. However, the recently developed phospholipid form of quercetin (Quercetin Phytosome^®^) has been found to increase oral bioavailability 20-fold, which is an invaluable improvement for quercetin, which is considered to be a phyto component capable of blocking the virus at every stage of the viral life cycle [328]. In addition, it has been suggested that hyperforin, which is the main polyphenol component of *Hypericum perforatum*, easily inhibits the proinflammatory effects of various cytokines at the 1.0 μM level in isolated rats and human pancreatic islets, giving the cells a long-term “cytokine resistance” [329]. In an in-silico study with rhoifolin, curcumin, (−)-epigallocatechin gallate, and scutellarin from other polyphenols, it has been calculated that these phytochemicals show very good binding affinities to the active site of 3CL^pro^ of SARS-CoV-2. Based on the values obtained, it has been suggested that these vegetative structures will create probable selective interactions with 3CL^pro^ [323]. While another member of the flavonoid family, hesperetin, demonstrated promising 3CL^pro^ inhibition in a cell-based cleavage assay [330], in an in vitro study, Sotetsuflavonen (biflavonoid IC_50_: 0.16 μM) isolated from *Dacrydium araucarioides* arose as the most potent natural blocking of the RdRp stage with its potential close to remdesivir (EC_50_: 0.07 µM) [322,324]. Additionally, in a molecular docking scan performed with remdesivir and ribavirin from the standard antiviral drugs for eight polyphenolic compounds, structures were evaluated according to their binding energies and (ΔG kcal/mol); Remdesivir (−8.51) > gallic acid (−7.55) > quercetin (−7.17) > caffeine (−6.10) > Ribavirin (−6.01) > resveratrol (−5.79) > naringenin (−5.69) > benzoic acid (−5.54) > oleuropein (−4.94) > ellagic acid (−4.59) sequence was obtained. In particular, gallic acid and quercetin have been suggested to exhibit drug-like properties with promising pharmacokinetic results with even higher binding affinity to SARS-CoV-2 RdRp than Ribavirin [331].

In another molecular docking study, interesting results were obtained against COVID-19 for saponins, which are known to strengthen the immune system. Accordingly, glycyrrhizin (saponin) extracted from the roots of the licorice plant (*Glycyrrhiza glabra* L., *Glycyrrhiza uralensis* Fisch. Ex DC.) binds with good affinity to the active site of ACE2, while it has been determined that glycyrrhizin derivatives exhibit anti-SARS-CoV activities at concentrations even lower than 10 µM [323]. Glycyrrhizin also showed a distinct interaction by making five separate hydrogen bonds with GLN 127, LYS 5, LYS 137, ARG 131, and TYR 239 amino acids in the main protease [17]. Glucoside-saikosaponin B2 (EC_50_ = 1.7 μM), found in many medicinal plants including *Heteromorpha* spp., *Bupleurum* spp., and *Scrophularia scorodonia*, shows the strongest activity against some human pathogenic viruses, while it was also revealed that this saponin structure blocks the viral penetration of HCoV-229E into host cells, in a dose- and time-dependent manner [323].

Another study showed that the terpene class cryptotanshinone isolated from *Salvia miltiorrhiza* effectively exhibited anti-TMPRSS2 activity at 0.5 μM in the androgen-sensitive human prostate (LNCaP) adenocarcinoma cell line [332]. Again, cryptotanshinone, tanshinone IIA, and dihydrotanshinone I (IC_50_: 0.8, 1.6, and 4.9 μM), which are tanshinones with abietan diterpene structure obtained from *Salvia miltiorrhiza*, exhibited strong inhibition against PL^pro^, which is responsible for the innate immune antagonist and proteolysis of the host [323]. Among the triterpenes with quinone-methide structure obtained from *Tripterygium regelii*, iguesterin and pristimerin are among the most potent 3CL^pro^ inhibitors. According to SAR analysis, the quinone-methide moiety is thought to be directly responsible for 3CL^pro^ inhibition [323].

Tannic acid and 3-isotheaflavin-3 gallate (obtained from *Camellia sinensis*) from the tannin class, which is known to protect cells from oxidative damage with its antioxidative properties, are other structures that show promising activity against 3CL^pro^ [17]. One group of secondary metabolites with a wide range of bioactivity is the alkaloids. Some studies with alkaloids that show activity against coronaviruses will now be summarized. The main active ingredient of *Carapichea ipecacuanha* roots, emetine (EC_50_: 0.30 μM for HCoV-OC43 and EC_50_: 1.43 μM for HCoV-NL63), has exhibited strong in vitro inhibition against different coronavirus replications [323]. Tryptanthrin, the major component in the leaf of *Strobilanthes cusia* plant belonging to the *Canthaceae* family, has interacted with the active site of HCoV-NL63 3CL^pro^ [330], and rhein and berberine found in Aloe vera (*Aloe barbadensis*) has shown strong binding affinities to the SARS-CoV-2 3CL^pro^ receptor [333]. Thymoquinone (ΔG: −5.5 kcal/mol) in the essential fixed oil of the *Nigella sativa* plant, which also has been previously reported to have antiviral activity against avian influenza virus (H9N2), is a bioactive structure that has shown good affinity for the ACE2 receptor of SARS-CoV-2 with effective binding energy [334,335].

**Table 2:** Potential natural products against COVID-19.

Phytochemical	Plant Molecule/ (Natural Source)	Mechanism of Action/(Experimental Results)	Refs
Flavonoids	Baicalin (*Citrus*)	ACE2 inhibitor of SARS in FRhK-4 cell line (IC_50_: 2.24 μM; EC_50_: 12.5 μg/mL)	[322]
		Binding with TMPRSS2 of SARS-CoV-2 (ΔG: −8.46 kcal/mol)	[324]
		3CL^pro^ inhibitor of SARS-CoV-2 on Vero E6 cells (IC_50_: 6.41 µM, EC_50_: 10.27 µM, SI: 19 µM)	
	Quercetin (Vegetables)	Binding with ACE2 of SARS-CoV-2 (ΔG: −8.66 kcal/mol)	
		3CL^pro^ inhibitor of SARS-CoV-2 (ΔG: −6.6 kcal/mol EC_50_ = 83.4 μM; CC_50_ = 3.32 μM)	[323]
	Sotetsuflavonen (*Dacrydium araucarioides)*	RdRp inhibitor of SARS-CoV-2 (IC_50_: 0.16 μM)	[324]
	Hesperetin (*Citrus*)	3CL^pro^ inhibitor of SARS-CoV-2 in a cell-based cleavage assay (IC_50_: 8.3 μM)	[330]
	Kaempferol (*Sambucus formosana* Nakai)	Binding with ACE2 and 3CL^pro^ of SARS-CoV-2 (ΔG: −7.20 kcal/mol) TMPRSS2 inhibitor of SARS-CoV-2 (ΔG: −7.80 kcal/mol)	[326]
	Rhoifolin (*Hypericum perforatum)*	Binding with 3CL^pro^ of SARS-CoV-2 (ΔG: −8.37 kcal/mol)	[323]
	Scutellarin (*Hypericum perforatum)*	Binding with of 3CL^pro^ of SARS-CoV-2 (ΔG: −8.32 kcal/mol)	
	Naringenin (*Citrus*)	Binding with RdRp of SARS-CoV-2 (ΔG: −5.69 kcal/mol)	[331]
Glycosylated flavonoids	Baicalein (*Curcuma longa* L.)	3CL^pro^ inhibitor of SARS-CoV-2 on Vero E6 cells (IC_50_: 0.94 µM, EC_50_: 1.69 µM, SI: 118 µM)	[324]
	Hesperidin (*Citrus*)	Binding with ACE2 protein of SARS-CoV-2 (ΔG: −8.3 kcal/mol)	[325]
Polyphenols	(-)-Epigallocatechin gallate (*Hypericum perforatum*)	Binding with 3CL^pro^ of SARS-CoV-2 (ΔG: −7.96 kcal/mol)	[323]
	Caffeic acid (*Sambucus formosana* Nakai)	ACE2 inhibitor of HCoV-NL63 (IC_50_: 8.1 μM)	
	Ellagic acid (*Berry*)	Binding with RdRp of SARS-CoV-2 (ΔG: −4.59 kcal/mol)	[331]
	Psoralidin (*Cullen corylifolium* (L.))	PL^pro^ inhibitor of SARS-CoV (IC_50_: 4.2 μM)	[327]
Polyphenols (tannins)	Tannic acid (*Camellia sinensis*)	3CL^pro^ inhibitor of SARS-CoV-2 (IC_50_: 3 μM)	[17]
	3-Isotheaflavin-3 gallate (*Camellia sinensis*)	3CL^pro^ inhibitor of SARS-CoV-2 (IC_50_: 7 μM)	
Diarylheptanoids	Hirsutenone (*Alnus japonica*)	PL^pro^ inhibitor of SARS-CoV (IC_50_: 4.1 µM)	[322]
	Curcumin (*Curcuma longa*)	PL^pro^ inhibitor of SARS-CoV (IC_50_: 5.7 μM)	
		3CL^pro^ inhibitor of SARS-CoV-2 (ΔG: −8.15 kcal/mol)	[323]
Prenylated phloroglucinol	Hyperforin (*Hypericum perforatum*)	3CL^pro^ inhibitor of SARS-CoV-2 (Inhibition of various cytokines at the 1.0 μM level in isolated rats and human pancreatic islets)	[329]
Glycosylated seco-iridoid	Oleuropein (*Olea europaea*)	RdRp inhibitor of SARS-CoV-2 (ΔG: −4.94 kcal/mol)	[331]
Alkylated chalcones	Isobavachalcone (*Cullen corylifolium* (L.))	PL^pro^ inhibitor of SARS-CoV (IC_50_: 7.3 μM)	[327]
Prenylated chalcones	Xanthoangelol E (*Angelica keiskei*)	PL^pro^ inhibitor of SARS-CoV (IC_50_: 1.2 μM)	
	Xanthoangelol F (*Angelica keiskei*)	PL^pro^ inhibitor of SARS-CoV (IC_50_: 5.6 μM)	
Saponins	Glycyrrhizin (*Glycyrrhiza glabra* L.)	Binding with ACE2 of SARS-CoV (ΔG: −9 kcal/mol)	[323]
		Binding with 3CL^pro^ of SARS-CoV (ΔG: −8.9 kcal/mol)	[17]
Terpenoids	Iguesterin (*Tripterygium regelii*)	3CL^pro^ inhibitor of SARS-CoV (IC_50_: 2.6 μM)	[323]
	Pristimerin (*Tripterygium regelii*)	3CL^pro^ inhibitor of SARS-CoV (IC_50_: 5.5 μM)	
	Saikosaponin B2 (*Heteromorpha* spp.)	ACE2 inhibitor of HCoV-229E (EC_50_ = 1.7 μM)	
Abietane diterpene	Cryptotanshinone (*Salvia miltiorrhiza*)	TMPRSS2 inhibitor in the LNCaP cells (IC_50_: 2.42 μM)	[332]
		PL^pro^ inhibitor of SARS-CoV (IC_50_: 0.8 μM)	[323]
	Tanshinone IIA (*Salvia miltiorrhiza*)	PL^pro^ inhibitor of SARS-CoV (IC_50_: 1.6 μM)	
	Dihydrotanshinone I (*Salvia miltiorrhiza*)	PL^pro^ inhibitor of SARS-CoV (IC_50_: 4.9 μM)	
Alkaloids	Emetine (*Carapichea ipecacuanha*)	3CL^pro^ inhibitor of HCoV-OC43 (EC_50_: 0.30 μM)	
		3CL^pro^ inhibitor of HCoV-NL63 (EC_50_: 1.43 μM)	
	Caffeine (*Cocoa* beans)	Binding with RdRp of SARS-CoV-2 (ΔG: −6.10 kcal/mol)	[331]
	Tryptanthrin (*Strobilanthes cusia*)	3CL^pro^ inhibitor of HCoV-NL63 (IC_50_: 1.52 μM; ΔG: −8.2 kcal/mol)	[330]
	Berberine (*Aloe barbadensis*)	Binding with 3CL^pro^ of SARS-CoV-2 (ΔG: −8.1 kcal/mol)	[333]
Anthraquinone	Rhein (*Aloe barbadensis*)	Binding with 3CL^pro^ of SARS-CoV-2 (ΔG: −8.9 kcal/mol)	
Quinone	Thymoquinone (*Nigella sativa*)	Binding with ACE2 of SARS-CoV-2 (ΔG: −5.5 kcal/mol)	[334]

Preclinical studies emphasize that the following home-based medicinal plants improve symptoms such as pulmonary fibrosis, lung damage, and organ failure based on sepsis, and improve lung function for patients who are severely infected with SARS-CoV-2: Curcumin, the bioactive compound in turmeric (*Curcuma longa*); *S*-allyl cysteine, allicin, and diallyl thiosulfonate (allicin) components in garlic (*Allium sativum*); Quercetin, apigenin, and selenium in onion (*Allium cepa*); Cinnamaldehyde, eugenol, and linalol in cinnamon (*Cinnamoni cortex*); Ascorbic acid, an immunomodulator in lemon (*Citrus lemon*); and medicinal fungi with immunomodulatory properties from mycelia of *Lentinula edodes* (Shiitake mushrooms). It has been suggested that these phytochemicals can mitigate the symptoms by blocking various macrophages and interferons that cause cytokine storms with their immune-stimulating effects on the severe inflammatory symptoms seen in COVID-19 [336,337]. Furthermore, in an in vitro study, it was specified that contents of capsules in traditional Chinese medicine (Lianhua Qingwen) showed antiviral and anti-inflammatory activity by inhibiting SARS-CoV-2 replication. Again, when the same capsules are combined with Ribavirin, Lopinavir/Ritonavir, and Umifenovir basic therapy, and administered to patients infected with SARS-CoV-2, it has been reported that many important symptoms are overcome in a remarkably short time [321]. When we look at the evidence we have, there are many promising but disorganized and unsubstantiated studies on natural products. All this intense data accumulation should be researched through systematic and analytical evaluations to provide concrete results. However, in the short term at least, they should be put into practice as complementary therapy to essential drug treatment.

In studies computationally conducted with herbs, it has been demonstrated that in particular, phytochemicals of the flavonoid, terpene, and alkaloid classes can strengthen immunity by inhibiting the virus at different stages. However, in addition to these computational screenings, most of the studies with plant extracts are in vitro intensive studies lacking standardization and analytical validation. In phytotherapy studies, to determine the effectiveness of these plants against COVID-19 standardization and verification of quality controls of extracts of plants selected according to the results of theoretical screening should be considered from the very beginning. The focus should then be on in vivo and clinical trials validating in vitro studies. Disconnected evaluations bring nothing but a waste of time and resources that can save human lives [338].

## 9. Conclusions

While the effects and mutation capacity of SARS-CoV-2′s unique replication mechanisms are alarming, the increase in the rate of the spread of the virus has worried us even more. Therefore, we hoped to review studies and results from different disciplines in the fight against COVID-19 up to now. Currently, immunotherapy treatments in the fight against diseases such as infections are important for creating an immune response and increasing immune system resistance. Researchers are making great efforts to develop the immunotherapeutic COVID-19 vaccine. However, there is also concern that the vaccines developed may be less effective against the variants. As with the influenza vaccine, developing pan-coronavirus vaccines to protect against a variety of virus mutants is still an important goal.

Similarly, researchers from all disciplines have not been able to demonstrate effective drugs against COVID-19, despite rigorous efforts and effective cooperation. The convergence of computer-based approaches can help speed the discovery process of COVID-19 drugs and vaccines, and so the data obtained by focusing on techniques such as molecular docking, molecular dynamics, and homology modelling should also be combined with in vivo and clinical studies. Considering the huge number of available compounds, computational methods are preferred methods because of their low-cost, rapid drug design.

Along with vaccines and immunotherapy, the development of repurposed drugs and new drugs for the treatment of COVID-19 is another global fundamental strategy. In particular, repurposed drugs can save time compared to a new drug-active molecule as they have passed clinical phase studies, safety profiles, formulation stages, and economic feasibility processes. Besides, these drugs may allow for the development of combination possibilities with new drug classes to offer more effective therapies. Nonetheless, all these opportunities should be applied to patients along with randomized and placebo-controlled clinical trials organized in a centrally organized manner. A valuable review on the subject showed the possible targets that could be used to beat the SARS-CoV-2 virus, which is guiding and stimulating medicinal chemists to synthesize new anti-COVID-19 molecules. At this stage, the toxicity of chemotherapeutic supplements, the possible complex reactions of the immune system against them, the complexities of large-scale production of vaccines that require sensitive production and storage systems, and their access to humans all seem to be challenging processes. Therefore, to fill the gaps that will arise and until the vaccine is administered and the discovery of synthetic drugs is concluded, it seems reasonable that natural products and herbal remedies that are highly tolerable and able to work together with the current clinical standard of care will play a role as prophylactics and adjuvants. However, at this stage, ethnopharmacological studies cannot offer analytical verification, and herbal formulation remains theoretical because optimized extractions, simplification of the chemical complexity created by metabolite diversity, and biological analysis are time-consuming. Again, the problems of efficacy trials, pharmacokinetic profile, bioavailability, safe doses, and application of different herbal combination therapies according to the stage of the disease should be resolved. If the in-silico, in vitro, in vivo, and clinical studies, which are now done in a disconnected way, are planned regularly and consistently as sequential, complementary studies, then inexpensive, large-scale, and easily applicable natural product-based therapies that will offer home-based simple solutions to the COVID-19 impasse are candidates. It is very important to keep drug interactions in mind when using these types of treatments. At the same time, since it is a SARS-CoV-2 RNA virus, it should also be taken into account that the mutation rate of the genome is fast and combination therapy should be recommended as in HIV infections.

With the advance of the pandemic, the economy of agriculture and aquaculture has declined significantly, negatively affecting millions of people worldwide. The epidemic that started in a food market has shown that the management of food processing and production policies should be revised. In order to reduce the spread of the pandemic and to prepare for new epidemics, new collaborations and action plans must be developed between scientists, technologists, governments, individuals, and the food sector around the world. In the next process, the importance of food bioactive compounds in reducing the risk of disease by strengthening the immune system should be emphasized. While raising public awareness about immune-enhancing functional foods, easy public access to these foods should also be provided. We think that the issues highlighted by this review will contribute to and direct future research on the subject.

## Figures and Tables

**Table 1:** A running list of repurposing drugs for COVID-19.

Agent	Chemical Structure	Classification	Approved for
Molnupiravir	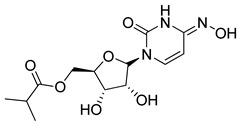	Antiviral	Phase 3 trial for COVID-19 treatment
Remdesivir	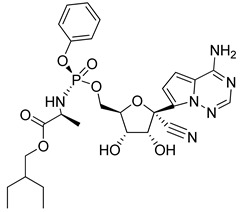	Antiviral	Treatment of COVID-19 and Ebola
Favipiravir	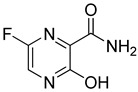	Antiviral	Treatment of Influenza
Chloroquine	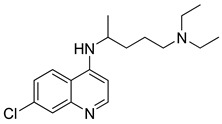	Antimalarial	Treatment of Malaria
Hydroxychloro-quine	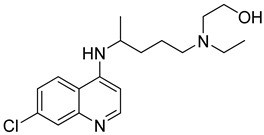	Antimalarial	Treatment of Malaria and some auto-immune diseases
Lopinavir	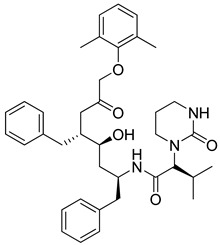	Antiviral	Treatment of HIV
Ritonavir	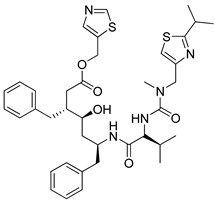	Antiviral	Treatment of HIV
Colchicine	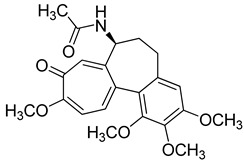	Anti-inflammatory	Treatment of familial Mediterranean fever (FMF) and acute gout flares
Naproxen	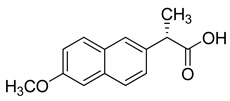	Anti-inflammatory	Treatment of acute gout, ankylosing spondylitis, bursitis, polyarticular juvenile idiopathic arthritis, osteoarthritis, tendonitis, rheumatoid arthritis, pain, and primary dysmenorrhea
Azithromycin	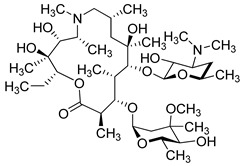	Antibacterial	Treatment of a number of bacterial infections.
Teicoplanin	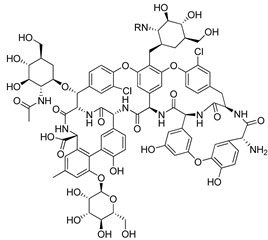	Antibacterial	Treatment of a number of Gram-positive bacterial infections and Ebola
Dexamethasone	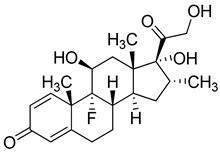	Corticosteroid	Treatment of a number of inflammatory conditions and for reducing the body’s immune response in the treatment of allergies and autoimmune diseases
Methylpredniso-lone	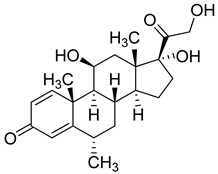	Corticosteroid	Treatment of allergic conditions, arthritis, asthma exacerbations, long-term asthma maintenance, acute exacerbation of multiple sclerosis
Nitazoxanide	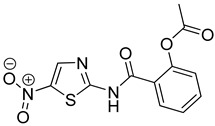	Antiparasitic	Treatment of diarrhea caused by *Giardia lamblia*
Ivermectin	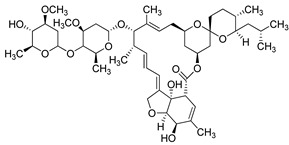	Antiparasitic	Treatment of parasitic infections such as intestinal strongyloidiasis and onchocerciasis
Camostat mesylate	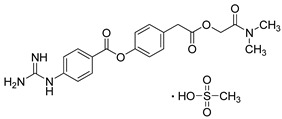	Anticancer	Treatment of chronic pancreatitis in Japan
Gemcitabine	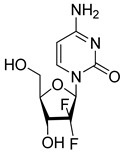	Anticancer	Treatment of a number of types of cancer
Imatinib	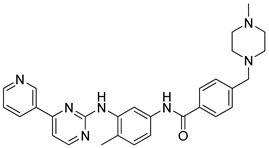	Anticancer	Treatment of a number of types of leukemia
Tamoxifen	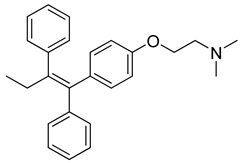	Anticancer	Treatment of breast cancer
Chlorpromazine	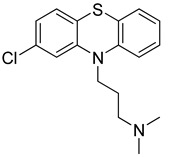	Antipsychotic and antihistamine	The management of Schizophrenia and other psychoses, mania and hypomania.In anxiety psychomotor agitation excitementNausea and vomiting
Fluphenazine	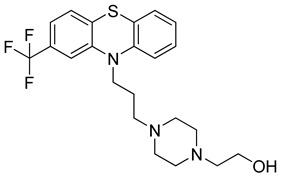	Antipsychotic and antihistamine	The management of Schizophrenia
Promethazine	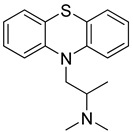	Antipsychotic and antihistamine	Treatment of allergic rhinitis, Vasomotor rhinitis. In addition to its antihistaminic action, it provides clinically useful sedative and antiemetic effects
Fluvoxamine	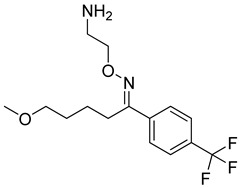	Antipsychotic	Treatment of depression, anxiety and other mood disorders
Losartan	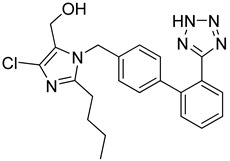	Antihypertensive	Treatment of high blood pressure
Metmorfin	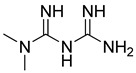	Antidiabetic	Treatment of type 2 diabetes

## Data Availability

No new data were created or analyzed in this study. Data sharing is not applicable to this article.
